# Previous Antibiotic Exposure and Antimicrobial Resistance Patterns of *Acinetobacter* spp. and *Pseudomonas aeruginosa* Isolated from Patients with Nosocomial Infections

**DOI:** 10.4274/balkanmedj.2016.1844

**Published:** 2017-12-01

**Authors:** Zorana M. Djordjevic, Marko M. Folic, Slobodan M. Jankovic

**Affiliations:** 1 Clinic of Control Hospital Infections, Kragujevac Centre Clinical, Kragujevac, Serbia; 2 Clinic of Pharmacology, Kragujevac Centre Clinical, Kragujevac, Serbia; 3 Kragujevac University School of Medicine, Kragujevac, Serbia

**Keywords:** Nosocomial infections, Pseudomonas aeruginosa, *Acinetobacter*, antimicrobial resistance

## Abstract

**Background:**

The alarming spread of antibiotic-resistant bacteria causing healthcare-associated infections has been extensively reported in recent medical literature.

**Aims:**

To compare trends in antimicrobial consumption and development of resistance among isolates of *Acinetobacter* spp. and *Pseudomonas aeruginosa* that cause hospital infections.

**Study Design:**

Cross-sectional study.

**Methods:**

A study was conducted in a tertiary healthcare institution in central Serbia, during the 7-year period between January 2009 and December 2015. The incidence rate of infections caused by *Acinetobacter* or *Pseudomonas*, as well as their resistance density to commonly used antibiotics, were calculated. Utilization of antibiotics was expressed as the number of defined daily doses per 1000 patient-days.

**Results:**

A statistically significant increase in resistance density in 2015 compared to the first year of observation was noted for *Acinetobacter*, but not for *Pseudomonas*, to third-generation cephalosporins (p=0.008), aminoglycosides (p=0.005), carbapenems (p=0.003), piperacillin/tazobactam (p=0.025), ampicillin/sulbactam (p=0.009) and tigecycline (p=0.048).

**Conclusion:**

Our study showed that there is an association between the resistance density of *Acinetobacter* spp. and utilization of carbapenems, tigecycline and aminoglycosides. A multifaceted intervention is needed to decrease the incidence rate of *Acinetobacter* and *Pseudomonas* hospital infections, as well as their resistance density to available antibiotics.

A substantial number of reports received from hospitals across the world is warning of the alarming spread of antibiotic-resistant bacteria causing nosocomial infections (NIs), which has led to increases in both costs and mortality (1,2,3). Clinically important pathogens such as *Acinetobacter* spp. and *Pseudomonas aeruginosa* are a great threat to affected patients due to their high rates of resistance and limited effective antimicrobial options. Both *Acinetobacter* and *Pseudomonas* are inherently resistant to many drugs, but can also become resistant to almost any antimicrobial agent. Resistance mechanisms in these bacteria involve beta-lactamase production, overexpression of multidrug efflux pumps and modifications of specific target sites or the outer membrane. Multiple drug resistance usually occurs as a result of a single potent resistance mechanism or the action of various mechanisms in a single isolate (4,5).

Previous studies have indicated the possibility of a causal link between the utilization of antibiotics and resistance among pathogens that cause NIs (6,7,8). However, the extent of the problem and its influence on health and healthcare costs remain unknown. Furthermore, in order to establish a firm hospital antibiotic policy, it is necessary to understand general resistance patterns and the local epidemiology. If at least one type of data is missing (e.g. utilization of antibiotics or resistance of isolates), this gap should be filled as soon as possible, in order to have all the necessary prerequisites for rational prescribing of antibiotics.

The main objective of our study was to compare trends in antimicrobial consumption and resistance development among isolates of *Acinetobacter* spp. and *P. aeruginosa* that cause NIs. A secondary objective was to explore whether the utilization of antibiotics and bacterial resistance were correlated.

## MATERIALS AND METHODS

A cross-sectional study was conducted during the 7-year period between January 2009 and December 2015 in a tertiary healthcare institution in central Serbia. This institution has medical and surgical specialties and four intensive care units for adults, and provides assistance to a population of 1.200.000 inhabitants. During the follow-up period, all patients older than 18 years who developed NIs caused by *Acinetobacter* spp. or *P. aeruginosa* of any localization were enrolled in the study. The exclusion criterion was the isolation of pathogens within the first 48 hours after admission to hospital. Also, if samples from the same location were microbiologically tested repeatedly during the same hospitalization, only the first isolate was taken into account for this analysis.

NIs were defined as infections that had not been present and without evidence of incubation at the time admission to hospital, and individual cases were classified as having NIs using the *Center for Disease Control* diagnostic criteria (9). NI monitoring took place as part of the integrated clinical survey of the patients every day, alongside daily review of the patients’ medical records and microbiological and laboratory data. All samples were sent to the Institutional Microbiology Laboratory, where microbial identification was carried out using conventional biochemical methods (10). Antimicrobial susceptibilities were determined using the disk diffusion method or Vitek-2 automated system (BioMerieux, France). The results were used to classify strains as susceptible or resistant according to the guidelines issued by *The Clinical and Laboratory Standards Institute* (11). Intermediately susceptible isolates were classified as resistant, according to the same guidelines.

Data on the utilization of antibiotics (oral and parenteral) were obtained from the hospital information system. The following antimicrobial groups according to the Anatomical Therapeutic Chemical classification (12) were analysed; J01CR05 and J01CR01 (combination of penicillins, including combinations of the beta-lactamase inhibitor; piperacillin/ tazobactam and ampicillin/sulbactam), J01DD (third-generation cephalosporins; ceftriaxone and ceftazidime), J01DH (carbapenems; meropenem and imipenem), J01GB (aminoglycosides; gentamicin and amikacin), J01MA (fluoroquinolones; ciprofloxacin and levofloxacin), J01AA12 (tigecycline) and J01XB01 (colistin). Antibiotic consumption was expressed in defined daily doses (DDDs) per 1.000 patient-days (PDs) for each prescribed antibiotic.

The study was approved by the Ethics Committee of the Clinical Centre Kragujevac (No: 01/43). Written informed consent was obtained from each patient before commencement of any procedure related to this research.

The data were analysed using descriptive statistics. The incidence density for each species was calculated as the ratio of the number of isolates and the total number of PDs in that year. The resistance density was calculated in the same way (as the number of resistant isolates and the total number of PDs) separately antibiotics, and then for antibiotic group. The number of PDs was obtained from the hospital’s administrative database. Linear regression was used for analysis of trends. The presence or absence of an association was tested using nonparametric Spearman’s rank correlation coefficient (r). The rate of change in the trends was calculated from linear regression as the percentage increase or decrease on an annual basis. Statistical hypotheses were considered true if the probability of the null hypothesis was less than 0.05. Analyses were performed using statistical software SPSS for Windows, version 18 (Chicago, IL, USA).

## RESULTS

During the study period 161.358 cultures were analysed, and 61.45% were positive for any microorganism; there were 968 isolates of *Acinetobacter* spp. and 447 isolates of *P. aeruginosa*, which caused NIs according to the defined criteria. The predominant anatomical localization of infection in both groups was the lower respiratory tract (48.54%), followed by surgical site, urinary tract and blood stream (33.36% vs. 8.54% vs. 5.92%, respectively), while other locations accounted for 3.60%. The difference between groups concerning the site of NIs was not statistically significant (p>0.05). The incidence densities of the isolated *Acinetobacter* spp. and *P. aeruginosa* (per/10.000 PD) during the period 2009 to 2015 are listed in Table 1. An increasing trend was recorded for the incidence density of isolates of both pathogens, but statistical significance was demonstrated only for *Acinetobacter* spp.

Isolates of *Acinetobacter* spp. showed a higher resistance density (per/10.000 PD) in 2015 compared to the first year of observation for the third-generation cephalosporins (12.54), aminoglycosides (11.43), carbapenems (11.4), fluoroquinolones (6.25), piperacillin/tazobactam (4.39), ampicillin/sulbactam, (2.46) and tigecycline (1.11) (Table 1). A statistically significant increase in resistance density was noted for third-generation cephalosporins, aminoglycosides, carbapenems, piperacillin/tazobactam, ampicillin/sulbactam and tigecycline (p<0.05). However, *P. aeruginosa* showed no significant trends for any of the antibiotics. Likewise the resistance density of *P. aeruginosa* to third-generation cephalosporins, aminoglycosides, carbapenems, fluoroquinolones, piperacillin/tazobactam, ampicillin/sulbactam and tigecycline was 4.66, 3.83, 2.75, 2.9, 0.41, 0 and 0.15 in 2015, respectively, being lower in comparison to that of *Acinetobacter* spp. The resistance density for colistin was less than 1.

Over the study period a trend for decreased total antimicrobial consumption was recorded (calculated as DDDs/1.000 PD), but without statistical significance. Table 1 shows that, in 2015, the highest utilization rate was recorded for third-generation cephalosporins, fluoroquinolones and aminoglycosides, accounting for 11.77%, 11.55% and 8.84% of total use, respectively. This was followed by carbapenems (4.59%), piperacillin/tazobactam (1.08%) and other antimicrobial drugs. Overall, there was a statistically significant increase in trends of antimicrobial consumption for carbapenems, tigecycline and colistin, while a decline in consumption was noted for aminoglycosides (p<0.05).

We observed a very strong correlation between the incidence density of *Acinetobacter* spp. and the consumption of tigecycline (r=0.821, p=0.023) and aminoglycosides (r=-0.857, p=0.014) (Table 2). Similarly, a very strong correlation was found between resistance density in *Acinetobacter* spp. and the use of carbapenems (r=0.786, p=0.036), tigecycline (r=0.955, p=0.001) and aminoglycosides (r=-0.856, p=0.014) (Table 3). There was no association between antimicrobial consumption and isolation or resistance rates for *P. aeruginosa*.

## DISCUSSION

Our study confirmed that *Acinetobacter* spp. and *P. aeruginosa* are becoming important opportunistic pathogens in our hospital, as reported in a previous study (13). During the observed period increasing trends in incidence density were observed for both pathogens, but the rate of change was higher for *Acinetobacter* spp. The results are highly consistent with recent studies conducted around the world (14,15).

In our study we used resistance density (per/10.000 PD) to describe the extent of resistance of isolates to antibiotics. This way of expressing the extent of resistance is more realistic than using just percentages, because the overall picture is less distorted when the samples are relatively small. The resistance densities of *Acinetobacter* spp. and *P. aeruginosa* to all tested antimicrobials were higher in 2015 in comparison to the first year of observation. Furthermore, the resistance of *Acinetobacter* spp. increased more rapidly, and the increasing trend in resistance density for third-generation cephalosporins, aminoglycosides, carbapenems, piperacillin/tazobactam, ampicillin/sulbactam and tigecycline was statistically significant. The exceptionally rapid increase in resistance to carbapenems is worrying, and new treatment strategies are necessary to fight against carbapenem-resistant *Acinetobacter* spp. *Acinetobacter* spp. display carbapenem resistance by producing different carbapenemase enzymes, but class B metallo-β-lactamases and class D oxacillinases are common as well (16). This pathogen is not especially virulent, but it produces infections that are accompanied by high mortality, especially in ICU patients, due to limited therapeutic options.

The rapid development of antimicrobial resistance of *Acinetobacter* spp. is likely to result from its ability to respond rapidly to challenges caused by antimicrobials through a variety of mechanisms of resistance. *Acinetobacter* is intrinsically resistant to many antibiotics and disinfectants because of the low permeability of its outer cell membrane and constitutive expression of certain efflux pumps, and it can also accumulate components of resistance mechanisms encoded on plasmids, transposons, and integrons from hospital settings associated with high antibiotic consumption. In addition, it is able to survive for prolonged periods in a hospital environment, potentiating its ability to cause outbreaks of infections with endemic resistant agents that spread horizontally in healthcare centres (17). These are all possible reasons for the proliferation of multidrug-resistant *Acinetobacter* spp. observed in our study. However, a similar increase in prevalence of such isolates has been observed all over the world, transforming this phenomenon into a global public health problem.

The resistance of *P. aeruginosa* to the tested antibiotics was subject to less pronounced changes during the study period, so the trend in resistance density lacked statistical significance. Stagnation or a slow increase in Pseudomonas resistance for the majority of tested drugs, except ciprofloxacin, was reported by Master et al. (18) for the period 1997 to 2009 in the USA. The susceptibility of Pseudomonas to tigecycline and ampicillin-sulbactam was not tested, because these bacteria have a natural resistance to these antimicrobials.

Clinical interest in colistin has been rising during the last decade due to the emergence of Gram-negative isolates that are resistant to carbapenems, cephalosporins and aminoglycosides and due to the scarcity of novel antibiotics. The high rates of resistance of *Acinetobacter* spp. and *P. aeruginosa* to common antibiotics (90-100%) in our hospital has put colistin in first place for the treatment of suspected NIs among critically ill patients. Even when a microbiological report on the sensitivity of the antibiotics is available, colistin remains the only optimal antibiotic in many patients. This resulted in a statistically significant increase in colistin utilization during the observed period (p=0.004). Frequent use of colistin is an indication of further loss of effective antimicrobial treatment options for Gram-negative bacterial infections. However, we still need to make additional efforts to ensure compliance with approved indications for colistin, in order to preserve the high sensitivity of Gram-negatives to this drug, which has already begun to decline in some countries (16,19,20).

Knowledge about antimicrobial consumption can be a valuable tool for healthcare providers and policy makers who are tasked with rationalizing the use of antibiotics. When consumption is expressed in standardized units of DDDs per 1.000 PD, it is possible to compare the quality of prescribing in different healthcare settings or regions (12). During the study period a decrease in overall utilization of antibiotics was observed (580.57 DDD/1.000 PD in 2009 vs. 529.18 DDD/1.000 PD in 2015), although without statistical significance. A previous study on 150 hospitals in France showed that median antibiotic use was in ranged from 60 DDD/1.000 PD in long-term care and mental health hospitals to 633 DDD/1.000 PD in university hospitals (21). Therefore, antibiotic use in our study did not deviate from that reported in other countries in Europe and the USA: 538 DDD/1.000 PD in Sweden; 609 DDD/1.000 PD in the Netherlands; 749 DDD/1.000 PD in Denmark; 764 DDD/1.000 PD in Ireland; and 789.8 DDD/1.000 PD in the USA (22). Our results are also in accordance with a recent meta-analysis of antibiotic consumption in acute care hospitals, encompassing 80 studies with data from 3130 hospitals between the years 1997 and 2013, that calculated that the mean consumption of all antibiotics was 586 DDDs/1.000 PD (95% confidence interval 540 to 632 DDDs/1.000 PD) (23).

In our study trends in the utilization of third-generation cephalosporins and fluoroquinolones did not either increase or decrease, while the utilization of aminoglycosides dropped significantly. This observation could be explained by the high rates of resistance of *Acinetobacter* spp. and *P. aeruginosa* to first-line antibiotics and the increasing incidence of multidrug-resistant agents. Switching to the prescription of second-line antibiotics, like beta-lactam/inhibitor combinations (piperacillin/tazobactam) and carbapenems, caused increasing trends in their utilization, which were statistically significant only for carbapenems (r2=0.666, p=0.025). Similar results were found in a 10-year (1999-2008) study by Goel et al. (24), in which trends in consumption were not significant for third-generation cephalosporins, fluoroquinolones or aminoglycosides. However, a significant increasing trend in consumption was seen for beta-lactam/inhibitor combinations (r2=0.45, p=0.033) and carbapenems (r2=0.68, p=0.022).

Although the contribution of tigecycline to total antibiotic utilization was not large (0.5% in 2015), its consumption increased significantly during the study period (from 0.27 DDD/1.000 PD in 2009 to 2.66 DDD/1.000 PD in 2015; p=0.001), probably due to the increased rate of multi-drug resistant Gram-negative isolates.

As expected from previous studies, there was a significant correlation between carbapenem use and the carbapenem resistance of *Acinetobacter* spp. in our study (r=0.786, p=0.036) (24,25). However, unlike in previous studies, we found a correlation between the development of resistance to tigecycline and its use (r=0.955, p=0.001) and the resistance of *Acinetobacter* to aminoglycosides and their use (r=-0.856, p=0.014). On the other hand, no association was found between *P. aeruginosa* and any of the antibiotics.

The reason why more significant correlations are missing is manyfold. The expression of antibiotic consumption using DDDs/1.000 PDs units is not a true reflection of individual exposure to antibiotics. Some patients are exposed to multiple broad-spectrum antibiotic drugs in the hospital surroundings, and these are mostly patients who are at high risk of acquiring infections by antibiotic-resistant pathogens. Furthermore, resistance to one antibacterial agent is frequently related to cross-resistance to other antibiotics. Cao et al. (26) demonstrated that consumption of carbapenems was associated with resistance to β-lactams, aminoglycosides and fluoroquinolones in *Acinetobacter* spp. Similarly, high resistance and overuse of antibiotics was reported for *P. aeruginosa* (27). Therefore, when there are both high consumption of antibiotics and high resistance rates, cross-resistance to all groups of antibiotics may occur, and in this manner mask the effect of the use of and resistance to a particular antimicrobial drug.

Finally, our study demonstrated the complexity of the process of resistance development, which has many influential factors; antibiotics as main promoters, inter-hospital transfer of patients, clonal widening of resistant microbes, dissimilar resistance mechanisms, healthcare system arrangements and infection control practices.

The results of this research provide the basis for implementation of corrective measures to improve the situation in terms of resistance among causative agents of NIs. Regardless of the existence of a NI department and an antibiotic policy, new and innovative measures are necessary. In the study setting, reports on the resistance of bacterial isolates to antibiotics and on the prescribing of antibiotics are sent to clinicians four times per year, to help them select the type and dosage of antibiotics. A well-established surveillance system is an essential component and the first step in an efficient fight against the increase in resistance among pathogens. Controls over the prescription of last-resort antibiotics have also been introduced by a commission consisting of infectious disease specialists, pharmacologists and intensive medicine specialists. Practical and effective measures in preventing the horizontal spread of pathogens are: a clean hospital environment and hand hygiene, which should be fully implemented. Taking into account the probability that only a few effective antibiotics for multidrug-resistant pathogens will be launched in the near future, the implementation of strategies against the development of acquired resistance will be essential (28). A harmonized approach (including antibiotics stewardship), based on evidence-based core strategies and specific planning associated with local aspects, should result in an improvement in the current situation. Nevertheless, there is no general agreement over which are the most credible measures for the control of resistance or what is the best combination of activities to reduce resistance (29).

Our study has several limitations:

1) Establishing a causal relationship between the rate of antimicrobial consumption and resistance rate may need other methodological approaches with more variables,

2) Possible confounders such as length of hospital stay, staffing level, case matching and hand hygiene compliance were not taken into consideration,

3) We were not able to genotype the isolates to identify clones,

4) Furthermore, this study was conducted in a single healthcare centre and thus the results cannot be generalized to other settings due to variations in medical practices.

In conclusion, our study describes trends concerning *Acinetobacter* spp. and *P. aeruginosa* in NIs. There was an association between the resistance density of *Acinetobacter* spp. and the utilization of carbapenems, tigecycline and aminoglycosides. However, in order to define the extent of the problem and to prepare guidelines for infection control, additional studies are necessary.

## Figures and Tables

**Table 1:**
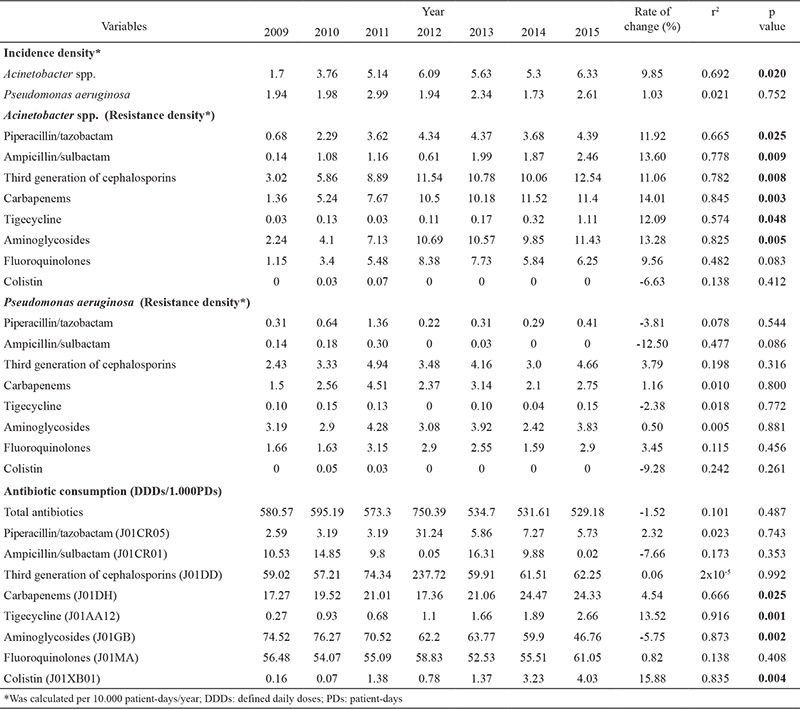
Isolation and resistance trends of *Acinetobacter* spp. and Pseudomonas aeruginosa and antimicrobial consumption in a tertiary healthcare institution in central Serbia, 2009-2015

**Table 2:**
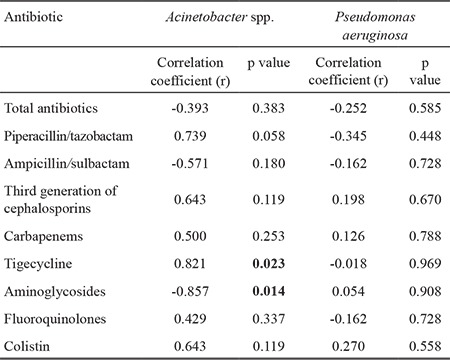
Spearman coefficient (r) of correlation between antibiotic consumption and incidence density (per/10.000 patient-days) for *Acinetobacter* spp. and Pseudomonas aeruginosa

**Table 3:**
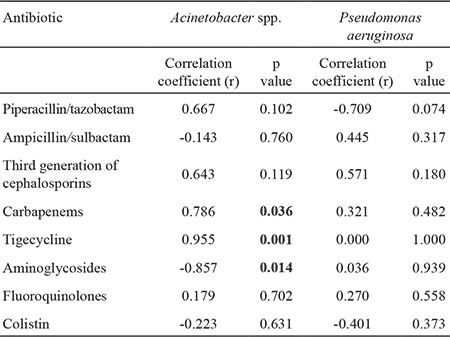
Spearman coefficient (r) of correlation between antibiotic consumption and resistance density (per 10.000 patient-days) for *Acinetobacter* spp. and Pseudomonas aeruginosa
